# Cortical spreading depression induces propagating activation of the thalamus ventral posteromedial nucleus in awake mice

**DOI:** 10.1186/s10194-021-01370-z

**Published:** 2022-01-24

**Authors:** Xiaoxi Fu, Ming Chen, Jinling Lu, Pengcheng Li

**Affiliations:** 1 Britton Chance Center and MoE Key Laboratory for Biomedical Photonics, Wuhan National Laboratory for Optoelectronics, Huazhong University of Science and Technology, Wuhan, China; 2 Research Unit of Multimodal Cross Scale Neural Signal Detection and Imaging, Chinese Academy of Medical Sciences, HUST-Suzhou Institute for Brainsmatics, JITRI, Suzhou, China; 3 School of Biomedical Engineering, Hainan University, Haikou, China

**Keywords:** Cortical spreading depression, Micro-endoscope, Thalamus, Migraine aura

## Abstract

**Background:**

As the relay centre for processing sensory information, the thalamus may involve in the abnormal sensory procedure caused by cortical spreading depression (CSD). However, few studies have focused on the transient response of thalamus during CSD. Our study aimed to investigate the neuronal activity of mouse thalamus ventral posteromedial nucleus (VPM) during CSD by in vivo micro-endoscopic fluorescence imaging of the genetic calcium probe GCaMP6s expressed in excitatory glutamatergic neurons.

**Methods:**

Thirty-four transgenic VGluT2-GCaMP6s mice were used in the experiments. An endoscope was inserted into the VPM for image acquisition. CSD was induced by KCl topically applied unilaterally on the cranial dura. Data were acquired in awake (ipsilateral or contralateral VPM, saline instead of KCl, MK-801 treatment) and anaesthetized (isoflurane, pentobarbital) states. Statistical analysis was performed using analysis of variance (ANOVA) by SPSS.

**Results:**

We found that after CSD induced in ipsilateral motor cortex, the neuronal activity increased and propagated from the posterior-lateral to the anterior-medial part of the VPM with an average speed of 3.47 mm/min. When CSD was induced in visual cortex, the response propagated in opposite direction, from the anterior-medial to the posterior-lateral part of the VPM. Aanaesthetics resulted in the suppression of VPM activation induced by CSD. No significant VPM activation was detected when CSD was induced in contralateral cortex or KCl was replaced by saline. When 5 mM MK-801 was applied to the dura, the electrode failed to record the DC shift of CSD, and there was no significant VPM activation after KCl application.

**Conclusion:**

CSD induced propagating activation of the ipsilateral VPM in awake mice. The response might correlate to the cortical location where CSD was induced and might be affected by anaesthetics. No significant VPM activation was detected in saline and mk801 experiment results indicated that this VPM activation is due to CSD rather than mouse motion or direct effect of the KCl applying to the intact dura. This finding suggests the potential involvement of thalamus in the migraine auras.

**Supplementary Information:**

The online version contains supplementary material available at 10.1186/s10194-021-01370-z.

## Introduction

Migraine is the third most prevalent neurovascular disorder worldwide [12]. Cortical spreading depression (CSD) is believed to be the pathophysiological correlate of migraine aura and headache attack [19]. The clinical manifestations of the gradual progress of aura symptoms are compatible with typical CSD [28]. Cerebral blood perfusion changes at a rate similar to the CSD as well [2, 14]. Recently, direct electrophysiological evidence of CSD has been detected in migraine patients with aura [17]. But the exact mechanisms of CSD in migraine remain under investigation.

CSD induce intense cortical neuronal excitation, reversible breakdown of ion homeostasis and prolonged depression of neuronal activity. It can activate meningeal macrophages [20], release ions (such as hydrogen, potassium) and neuropeptides (such as CGRP), and lead to instant or prolonged activation of dural nociceptors and trigeminal nucleus caudalis (TNC) [32, 33]. CSD leads to c-Fos expression in the amygdala as well as thalamic reticular nucleus (TRN), and the presence of the CSD wave in TRN was demonstrated using electrophysiology [25]. CSD can also induce changes in the cortical plasticity [26] and bilateral sensory disturbance [15]. As the critical sensory relay centre and high-order nuclei of nociceptive afference, the ventral posterior thalamus (VPM) has synaptic connections to the somatosensory cortex and TNC [22]. CSD-induced abrupt cortical turbulence may also influence VPM. However, few studies have focused on the transient response of thalamus during CSD, and there is a lack of evidence that CSD can induce transient activation in the VPM.

In this study, we focused on investigating how the thalamus ventral posteromedial nucleus (VPM) responds to CSD induced at mouse cortex using micro-endoscopy fluorescence imaging, which offers the opportunity to reach deep structures via miniaturized objective lens inserted into the brain, and achieves cellular-level time-lapse fluorescence detection [3]. We also investigated the effect of different anaesthetics on the VPM response. Glutamatpergic synaptic transmission is an important element in circuits between the thalamus and the cortex [9]. Vesicular glutamate transporter 2 (VgluT2) is responsible for trafficking and regulating the release of glutamate and has been detected in several thalamic nuclei, including the lateral geniculate complex, the posterior complex, the ventral posterolateral nucleus, the ventral anterior-lateral complex, the ventral medial nucleus and the VPM [4], which makes it suitable for our study.

## Materials and methods

### Fluorescence imaging system

**Fig. 1:**
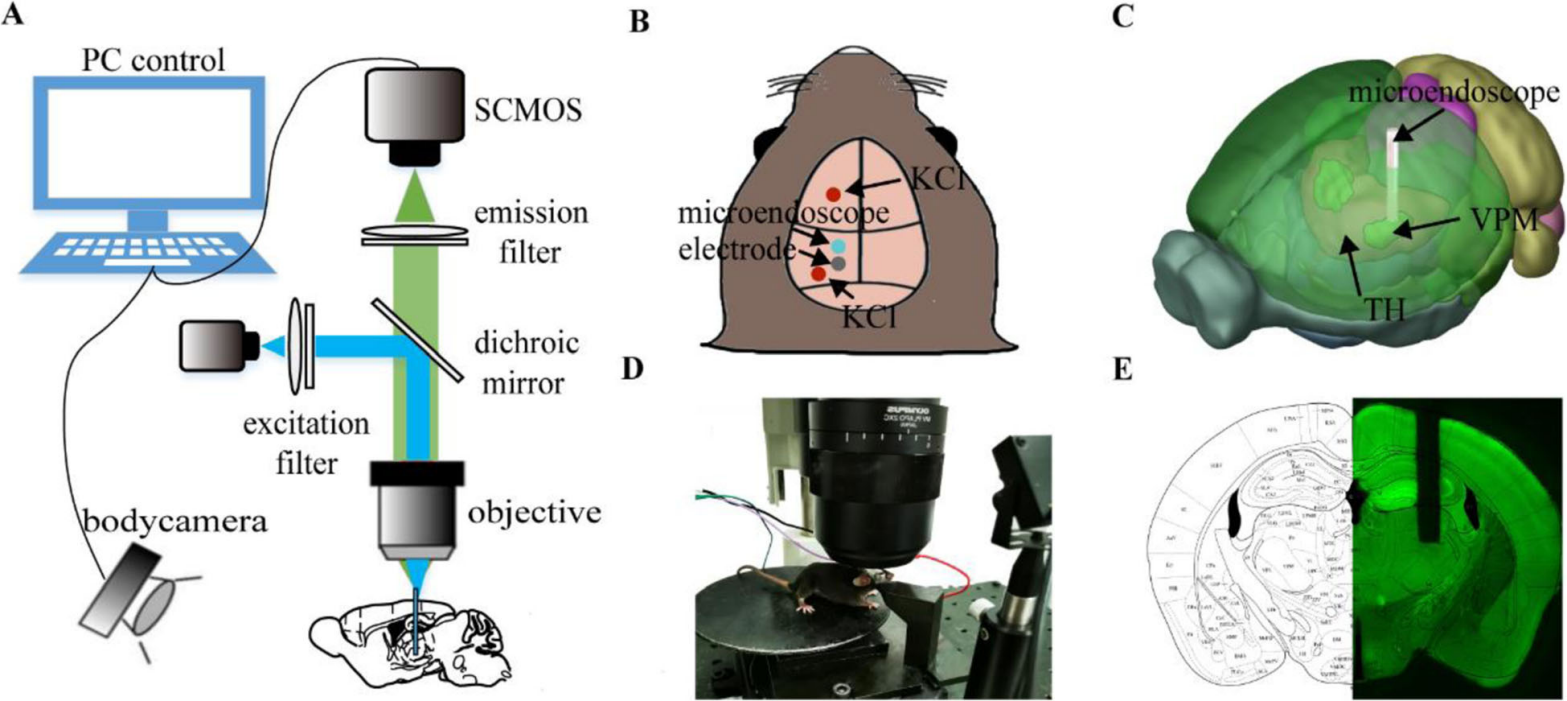
Optical system for VPM fluorescence imaging in awake animals. **A** Schematic of the imaging system. **B-C** Schematic of implantation of the GRIN lens and chronic electrode. The 3D model was obtained from the Allen brain atlas. **D** Head-fixed setup with an infrared camera for motion detection. **E** Brain slices matching the standard mouse brain atlas

We used an Olympus MVX10 microscope and a Hamamatsu ORCA Flash 4.0 SCMOS (2048 × 2048, pixel size 6.5 μm, 16 bit) for fluorescence imaging. The images were acquired by HCImageLive software. A high-power mercury lamp (U-HGLGPS, Olympus, Japan; 130 W) served as the light source. The GFP filter cubes contained a 460–490 nm bandpass filter, a 520 nm high pass filter, and a 500 nm dichroic filter (Fig. 1A). Fluorescence images were acquired using a 200 ms exposure time with 1024 × 1024 pixels array, amplified at 12.6×, and were saved by 2 × 2 spatial binning. Direct current (DC) potentials were filtered at 0–100 Hz, amplified at 50× and acquired at a sampling rate of 200 Hz through a differential amplifier (Model 3000, A-M system, USA). Mice were trained to habituate to the head-fix setup with an adjustable running wheel, as shown in Fig. 1D. A near infrared camera was equipped to monitor mouse movement.

Temporal synchronization of all data streams (fluorescence imaging, DC recording, body tracking camera) was achieved by the LabVIEW (National Instruments, USA) control program.

### Animal preparation

34 transgenic VGluT2-GCaMP6s mice weighing 27 ± 5 g (8–16 weeks old) were used in this study. Mice were acquired by hybridizing Vglut2-ires-Cre mice (Stock No. 028863) and Cre-dependent GCaMP6s mice (Ai96, Stock No. 028866) from the Jackson Laboratory. Mice were housed under a normal 12 h light/dark cycle with food and water provided ad libitum.

We used a Gradient Index (GRIN) lens (NEM-050-06-08-520-S-1.5p, Grintech, Germany; Single, 1×, NA 0.5, 0.5 mm in diameter) to collect the fluorescence signal (Fig. 1C). This GRIN lens is a cylindrical optical lens with a radial negative gradient of refractive index. It allows continuous refraction of light transmitted along the axial direction, achieving a smooth and continuous convergence of the light to a single point, which has the characteristics of focusing and imaging. The GIRIN lens would focus mercury lamp illumination near the top of the lens plane, relay the illumination into the brain, and relay neuronal signals of VPM to the lens plane above the cortex for image acquisition. Although glial activation around the implanted GRIN lens was inevitable like any other implanted entity such as electrodes, the optical penetration was up to ~ 650 μm, which could reach tissue lying beyond the glial activation layer for imaging [3].

Mice were anaesthetized with 2% isoflurane and placed in a stereotaxic apparatus during surgery. Body temperature was maintained at 37 ± 0.5 °C using a heating pad. After the skull was exposed and cleaned with saline, a craniotomy was performed (0.6 mm in diameter) at 1.7 mm posterior and 1.75 mm lateral to bregma. To prevent mechanical compression and damage, a cylindrical column of brain tissue above the VPM structure was removed by aspiration using a 27-gauge blunt needle [3]. The GRIN lens was fixed to a micromanipulator (MX7600L, Siskiyou, USA) and inserted into the exposed areas slowly to minimize bleeding and mechanical damage (150 μm/min). Two additional craniotomies were performed to implant electrodes. The recording electrode was implanted on the dura about 0.3–0.5 mm posterior to the GRIN lens. The recording electrode was made of silver wire (0.2 mm in diameter, CAS 7440-22-4) and attached to the dura. A steel screw (0.5 mm in diameter) was used as the reference electrode and implanted 1.5 mm deep into the nasal bone. In some experiments, electrodes were also implanted on the dura overlying the contralateral homologous cortex to simultaneously monitor electrophysiological signals in both hemispheres of the brain. We applied dental cement around the implants for stabilization and attach a metal head bar for head fixation, while a piece of flexible tube was affixed over the GRIN lens to protect it.

Mice were allowed to recover for 4 weeks before the experiment. Tolfedine (0.2 mg/kg, intraperitoneal) and penicillin (20,000 unit, intraperitoneal) were administered to minimize tissue swelling and inflammation for 3–7 days. Mice were habituated to the behaviour stage for several days before the CSD experiments.

### Experiment procedure

we applied 1 μl KCl solution (1 mol/L) on the dura to generate CSD. The occurrence of CSD was assessed by continuous recording of the DC potential. Mice were anaesthetized with 2% isoflurane and a craniotomy (0.5 mm in diameter) was performed to expose the dura overlying the motor cortex (AP, 2.5 mm; L, 2 mm) or visual cortex (AP, − 3.5 mm; L, 3 mm) (Fig. 1B) for KCl application 1.5 h before the start of the experiment. The dura remained intact. We generally recorded resting-state fluorescence images for 2–5 min before each CSD implementation. We induced CSD in the ipsilateral (*n* = 50) and contralateral hemisphere (*n* = 15) to determine the effect of CSD on the bilateral VPM of the thalamus in awake mice. For the experiments investigating the effect of anaesthesia on VPM activation, two common anaesthetics were used. The concentration of isoflurane (0.6%, 0.9% and 1.2%, *n* = 10 respectively) was controlled by an anaesthesia machine (VIP 3000 Veterinary Vaporizer, Midmark, USA). Pentobarbital (*n* = 13) was administered intraperitoneally at a dosage of 50 mg/kg. To determine whether CSD caused the activation of the VPM, saline was used to replace KCl (*n* = 16), or 5 mM MK-801 (*n* = 8) was administered to the dura overlying the motor cortex ipsilateral to the GRIN lens 1 h before KCl application to block CSD induction. Fluorescence images were acquired at 5 Hz for 10 min at each CSD experiment, and mice were allowed to recover for more than 1.5 h before the next CSD stimulation to avoid the influence of previous CSD. No more than five CSDs were induced per mouse per day. In our experiments, it takes us at least one month waiting for a mouse to recover from the surgery of GRIN lens implantation. Furthermore, the mouse cannot be used if the inflammatory response due to cellular edema or necrosis blurs the fluorescent signal. These difficulties in the preparation of the animal models limit the total number of animals can be used in our study so that we have to carry out multiple KCl applications in the same animal. Under the condition of isoflurane with three different anesthetic level, the sequence of anesthetic level applied is randomized for each successive KCl application in the same mouse to reduce the carry-over effect by repeated measures.

### Data processing

We performed data processing using code written in MATLAB R2018b. We derived the fluorescence intensity change (△F/F) by calculating $\Delta  F/F=\frac{F(t)-{F}_0}{F_0}$, where F(t) is the instant ROI fluorescence intensity and F_0_ is the average ROI fluorescence intensity among the resting-state recordings. Statistical analysis of experiments (involving ipsilateral and contralateral VPM in awake state, saline application, pentobarbital anesthetization and MK-801 treatment) was performed using the analysis of variance (ANOVA) between CSD stimulated state and resting state. The repeated measurements of 3 levels of isoflurane and awake state in each mouse were analyzed with the repeated measures ANOVA. A *P* value less than 0.05 was considered significant. Data are presented as the mean ± standard deviation (SD). Sample size was calculated by PASS (α = 0.05, power = 90%) with estimated mean value of 0.2 and SD value of 0.08 for ipsilateral fluorescence intensity change. Resting state fluorescence change were estimated with mean value of 0.03 and SD value of 0.08. The calculation results indicated that the sample size greater than 6 could be accepted. No data were excluded except when the micro-endoscope was inserted into the wrong region or inflammatory tissue obstructed imaging severely.

For mouse motion detection, the global difference was used to measure the amplitude of movement, which was defined as $\varDelta {I}_m=\frac{1}{N_{count}}\sum \left({I}_{t+1}-{I}_t\right)$, where I_t_ and I_(t + 1)_ are two contiguous images acquired by a body tracking camera and N_count_ is the total number of pixels of each image, which was 640 × 480 in our experiment.

### Confirmation of the implanted micro-endoscope locations

**Fig. 2:**
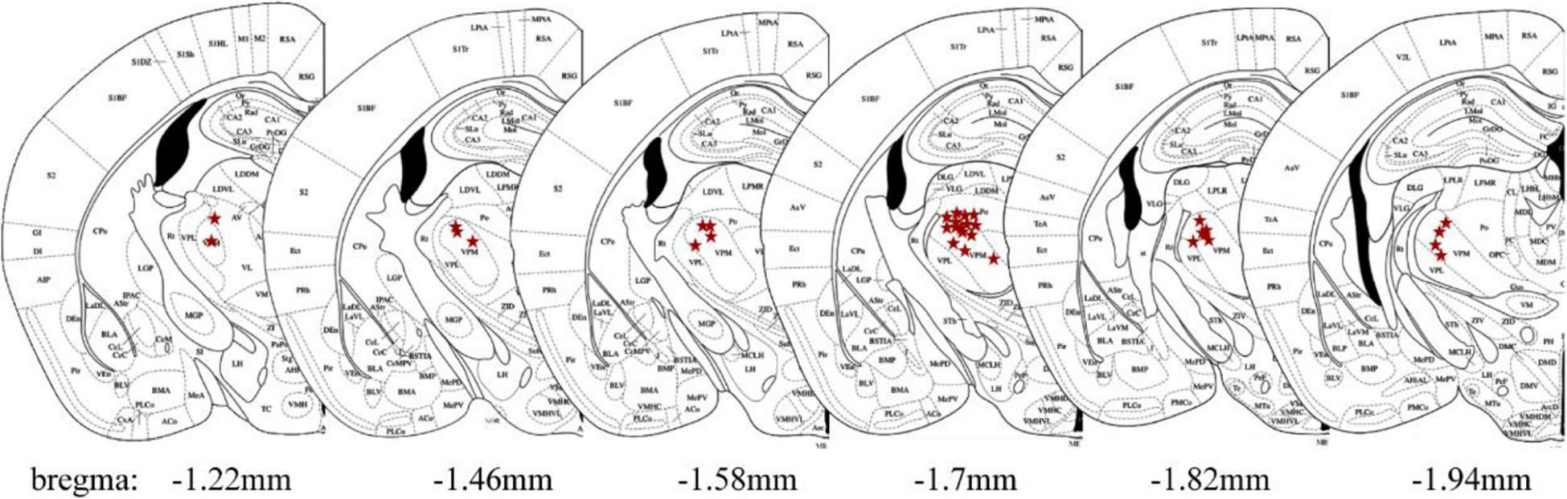
Locations of all the implanted GRIN lenses. The red star symbols indicated the micro-endoscope insertion point of 34 mice in our experiment, matching the standard mouse brain atlas

Mice were sacrificed and perfused transcardially with 4% paraformaldehyde solution (PFA). Coronal sections (100 μm thick) of the brain around the micro-endoscope implantation sites were prepared with a vibratome. Brain slice fluorescence images were acquired with a microscope (MVX10, Olympus, Japan). The edge contour of the hippocampus was extracted from each image of the brain slices and registered to the reference brain atlas by manual translation, scaling, rotation and skewing. This deformation operation was applied to the whole slice for overall registration, and the micro-endoscope insertion point was located (see Fig. 1E and Fig. 2).

## Results

**Fig. 3:**
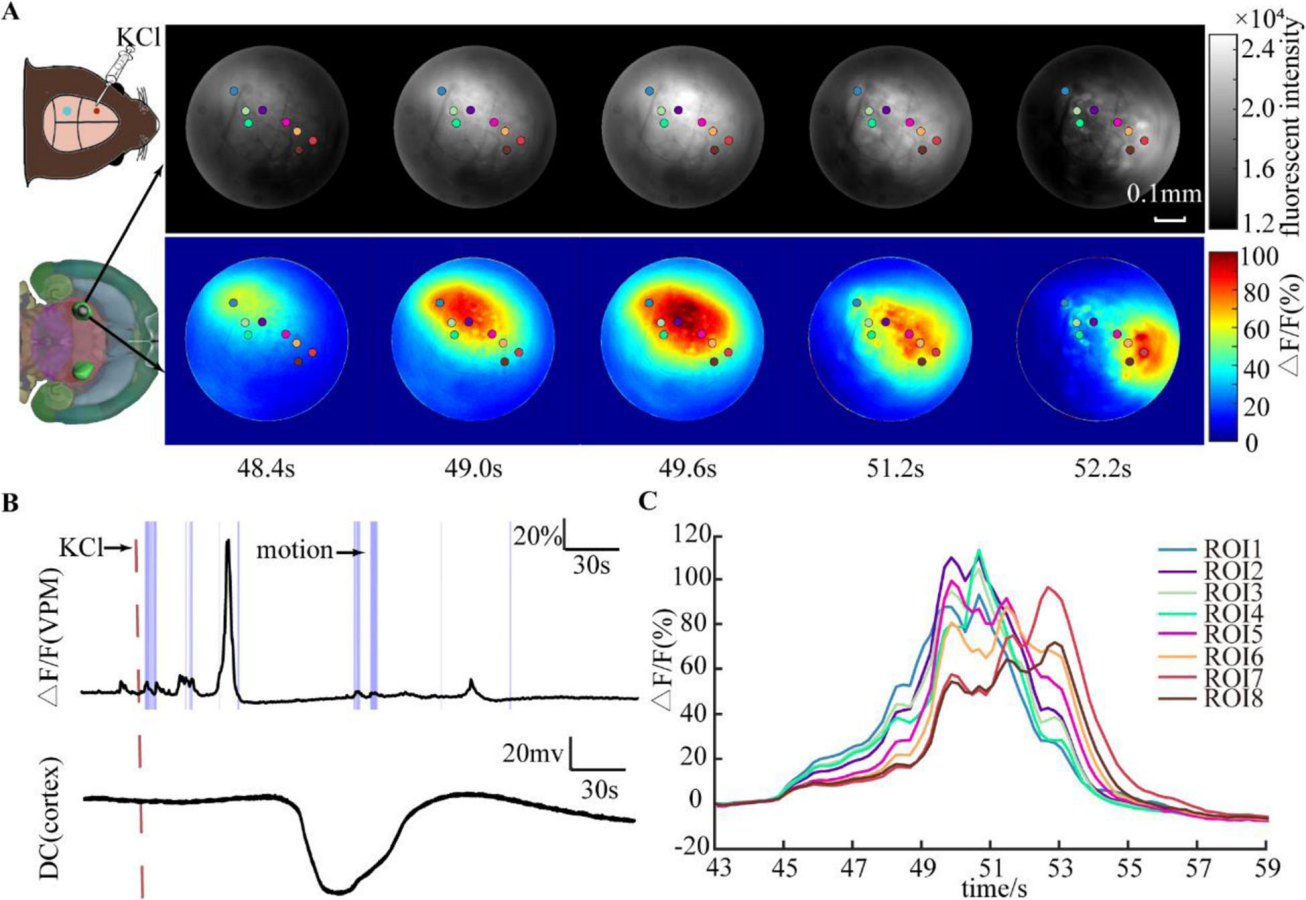
Spatiotemporal evolution of CSD-related responses from the VPM in a single trial. **A** Fluorescence response evoked by one CSD. Eight ROIs were selected to analyse their temporal-spatial characteristics. The upper row showed the raw fluorescence intensity, and the lower row showed the relative fluorescence change. The response first arisesd at the upper left and ends at the lower right of the imaging field. **B** The fluorescence intensity changes of the VPM and the simultaneous DC shift in the cortex. Dashed line indicated the timing for adding KCl. The purple shaded areas indicated the movement of the mouse. The motion-related fluorescence change was relatively small compared to that of CSD. **C** The fluorescence intensity changes of the eight ROIs. The ROI located more posterior- lateral reached the maximum fluorescence intensity earlier than the other ROIs

**Fig. 4:**
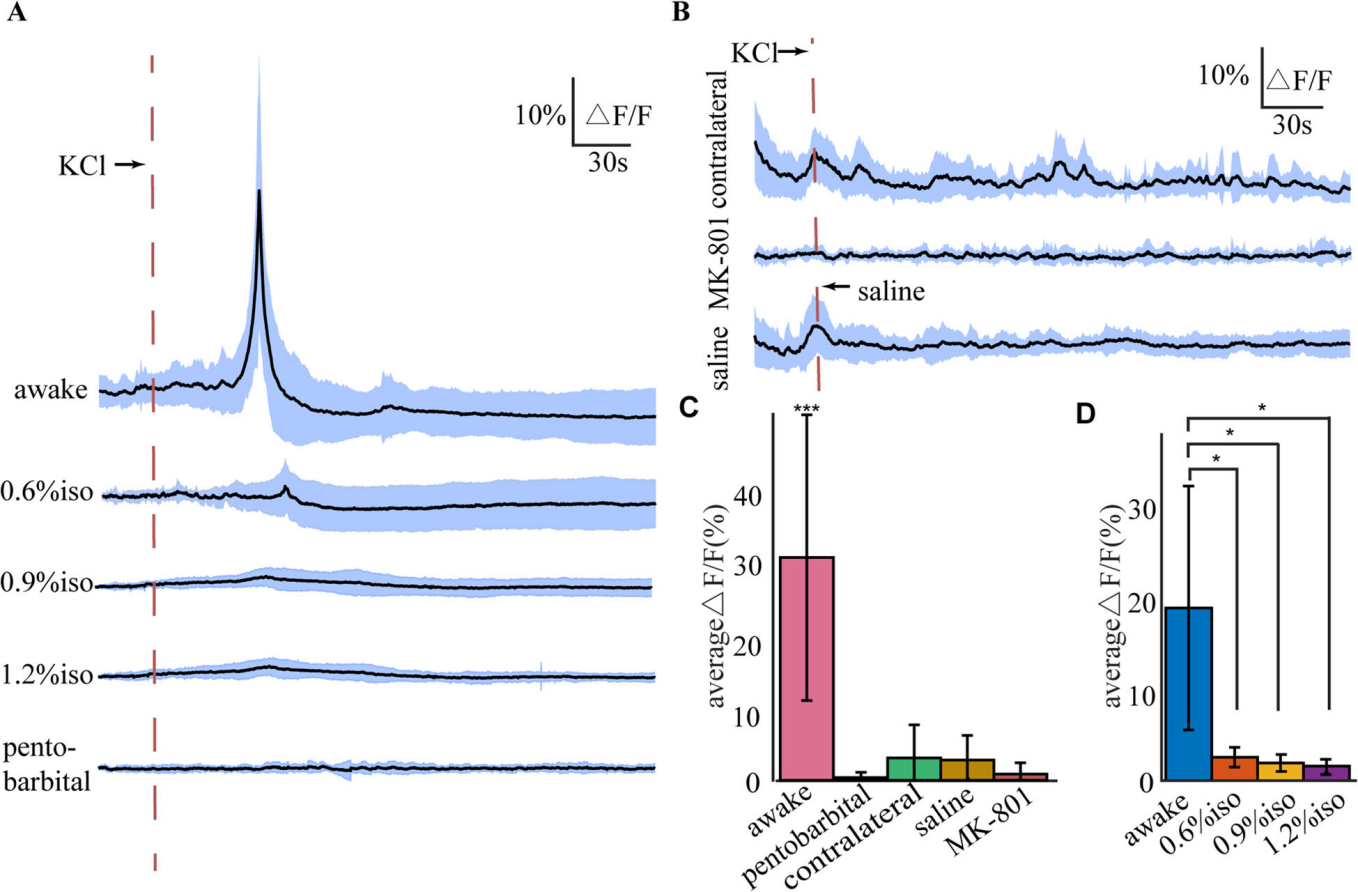
Statistics of KCl-induced responses in the VPM under different conditions. **A** KCl application activated VGluT2 neurons in the ipsilateral VPM in awake mice (*n* = 50). When anaesthetized with isoflurane (0.6%, 0.9%, 1.2%, *n* = 10 respectively), or 50 mg/kg pentobarbital (*n* = 13), CSD did not significantly activate VGluT2 neurons in the VPM. Data are presented as means ± SD. **B** KCl application did not activate VGluT2 neurons in contralateral VPM (*n* = 15), or when the cortex was topical affected by NMDA receptor antagonist MK-801 (*n* = 8). When saline was applied ipsilaterally instead of KCl (*n* = 16), there was no significant activation. Data are presented as means ± SD. **C** The maximum average relative fluorescence change under different conditions. Data are presented as means ± SD. ***P<0.001 based on ANOVA between stimulated state and resting state in each case. **D** The maximum average relative fluorescence changes of 10 experimental mice under isoflurane anesthetized or awake state. Data are presented as means ± SD. *P<0.05 based on repeated measures ANOVA between awake state and 3 different levels of isoflurane anesthetized state. When anaesthetized with isoflurane, the activation of VGluT2 neurons in the VPM by CSD application was significantly decreased (*P*<0.05)

A total of 50 CSDs were induced to investigate ipsilateral VPM activity during CSD when mice were awake. We applied 1 mol/L KCl solution to the dura overlying the ipsilateral motor cortex (AP, 2.5 mm; L, 2 mm) to induce CSD on awake mice. As shown in Fig. 3A and additional file 1, the increase in neural activity in the VPM induced by CSD showed a “propagating-like” spatial pattern that spread from the posterior-lateral to the anterior-medial side of the VPM. We randomly selected eight points with significant cellular activation as the regions of interest (ROIs). The fluorescence in the ROIs located on the posterior-lateral side reached its maximum intensity earlier than that in the ROIs located on the anterior-medial side (Fig. 3C). The purple shaded areas indicated in Fig. 3B represent the period of movement of the mouse obtained through a motion monitoring system. Mouse movement can lead to the activation of VGluT2 neurons in the VPM, but the change in fluorescence intensity was smaller than that induced by CSD. We observed that the VPM was significantly activated by CSD induced at cortex while there was no movement of mice. After application of KCl, the intensity of GCaMP6s fluorescence within the field of view started to increase at 39.72 ± 11.11 s (see Fig. 4A) and reached a peak at 56.40 ± 9.55 s, with an amplitude of change in fluorescence (△F/F) of 31.52 ± 20.23%. We estimated an average propagating speed of 3.47 mm/min.

We also conducted 15 trials investigating the effect of CSD on the contralateral VPM of the thalamus. No significant neural activity of VGluT2 neurons was observed in the VPM after CSD induction in the contralateral cortex (Fig. 4B and C, *P* = 0.3974). In the contralateral VPM, the changed signal was highly correlated with the motion of mice (see Additional file 2). These results suggested that the effects of CSD are unilateral in the thalamus.

To determine the observed neural activity was caused by CSD but not by the direct effect of the KCl applying to the intact dura, we conducted two experiments: 1) applying of saline (*n* = 16) instead of KCl; 2) topical application of the N-methyl-D-aspartate receptor antagonist MK-801 (*n* = 8) to block the induction and propagation of CSD [6]. The results showed that there was no significant activity of VGluT2 neurons after saline application (Fig. 4B and C, *P* = 0.1225). The activity of VPM when saline was applied was correlated with mouse motion. This motion could be that the mice got nervous from the proximity of microinjector. When 5 mM MK-801 was topically applied to the cortex with intact dura, the electrode failed to record the typical DC shift of CSD, and there was no significant neural activity in the VPM after KCl application (Fig. 4B). These results suggest that KCl-induced CSD triggers the VPM response.

**Fig. 5:**
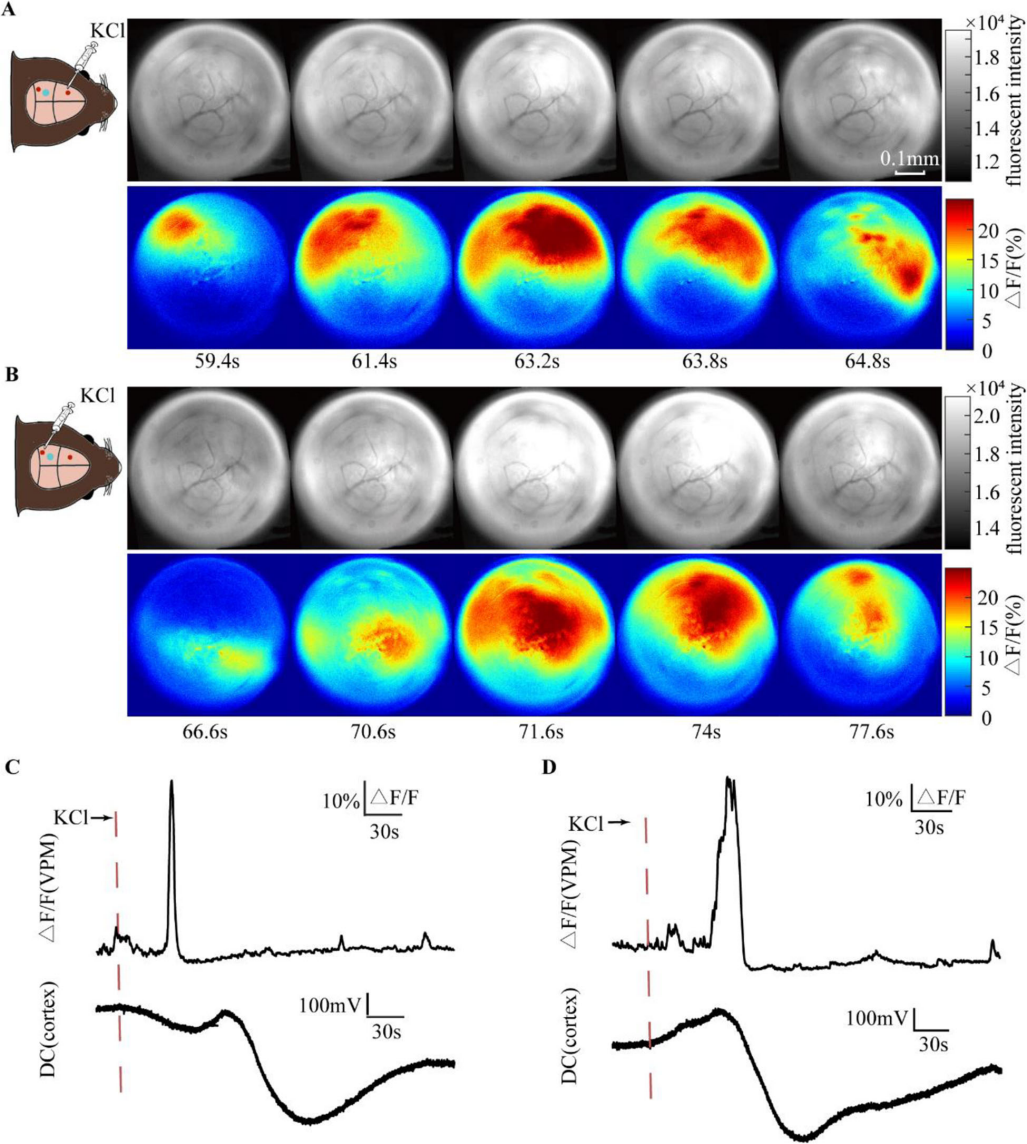
Different propagating directions of VPM responses when CSD was induced in motor cortex and visual cortex respectively. **A** When CSD was induced in motor cortex, the response propagated from the posterior-lateral to the anterior-medial side of the VPM. **B** In the same mouse, when CSD was induced in visual cortex, the response propagated in opposite direction, from the anterior-medial to the posterior- lateral side of the VPM. **C** The fluorescence intensity changes of the VPM and the simultaneous DC shift in the cortex when CSD was induced in motor cortex. **D** The fluorescence intensity changes of the VPM and the simultaneous DC shift in the cortex when CSD was induced in visual cortex

On four of the mice, another craniotomy was performed to expose the dura overlying the ipsilateral visual cortex (AP, 2.5 mm; L, 2 mm) to induce CSD. As shown in Fig. 5, the response propagated in opposite direction, from the anterior- medial to the posterior-lateral side of the VPM when CSD was induced in visual cortex. VPM has projection relationships mainly with the somatosensory cortex. Different directions of CSD had different temporal sequences of effects on somatosensory cortical subregions, which was similar to the different propagating directions of VPM responses in our result. This suggested that the propagation of the VPM activation may be related to the propagation of CSD in the cortex.

We investigated KCl-induced VGluT2 neurons responses in the VPM under anaesthesia. We applied isoflurane (0.6%, 0.9%, 1.2%, *n* = 10 respectively) to assess how the anaesthesia concentration regulated the effects of CSD on the VPM. When anaesthetized with 0.6% isoflurane, at which concentration mice were so slightly anaesthetized that they could still move, the VPM response decreased distinctly to 2.728 ± 1.146% compared with the 20.133 ± 14.232% of the fluorescence change when these 10 mice were in awake state (Fig. 4A and D, *P* = 0.027). When the concentration of isoflurane was increased to 0.9% and 1.2%, the response decreased 2.054 ± 0.990% (*P* = 0.025) and 1.692 ± 0.786% (*P* = 0.025) accordingly. However, there showed no significant difference in VPM fluorescence change under different isoflurane level (Fig. 4D, *P* = 0.087 between 0.6% and 0.9%, *P* = 0.096 between 0.6% and 1.2%). Due to the influence of isoflurane on the transfer of CSD to the thalamus [16, 24], we also used 50 mg/kg pentobarbital for anesthetization (*n* = 13), and CSD did not arouse a distinct response either (Fig. 4A and C, *P* = 0.5512).

To investigate the influence of multiple applications of KCl in the same animal, we picked out the data of first CSD induction in each mouse, including awake (*n* = 10), contralateral (*n* = 4), isoflurane anaesthetized (*n* = 4 for each concentration), pentobarbital anaesthetized (*n* = 4), MK-801 treatment (*n* = 3) and saline application (*n* = 3). The results also suggested that CSD can induce activation only in the ipsilateral VPM, and the use of a small amount of anaesthetic leaded to the suppression of VPM activation (data not shown).

## Discussion

Our study demonstrates for the first time that CSD can induce propagating-like neuronal activation in the ipsilateral VPM in awake mice by using in vivo micro-endoscopic imaging of a calcium probe. The neuronal activity of glutamatergic neurons in the VPM started to increase at 39.72 ± 11.11 s after KCl application on dura overlying motor cortex and propagated from the posterior-lateral to the anterior-medial part of the VPM, with an average speed of 3.47 mm/min. The propagating direction depended on the cortical location where CSD was induced. Previous studies report that KCl typically evokes multiple CSD waves. In our study, CSD was induced by application of 1 μl KCl (1 M), which usually evoked one CSD wave per application in our preliminary experiments in anesthetized mouse. In awake mice, multiple CSD waves were observed in 22 trials of total 50 trials using this amount of KCl. It is worthy to note that the subsequent CSD waves did not activate distinct VPM activation expect for the first wave. Future investigation is needed to study the mechanism underlying this different activation of thalamus caused by the first cortical CSD wave from that caused by the subsequent CSDs.

Our experiments indicated that the movement of mice did not fundamentally affect the activation results. The motion-related signal change was relatively small compared to that of CSD, and highly coincident with the motion of mice, which is easy to distinguish. The detected fluorescence signals increased significantly only in awake mice but not in anesthetized mice, confirming that the signal changes were VPM neural related but not produced by the external fluorescence interference such as activation of cortical neurons by CSD, as the use of isoflurane and pentobarbital will not block the induction and propagation of the CSD [23]. The experiment of using NMDA receptor antagonist MK-801 to inhibit CSD induction showed no significant activation of the VPM, which suggested that this activation was induced by CSD rather than direct stimulation of the dura by KCl.

In our experiment results, the use of a small amount of anaesthetic resulted in the suppression of VPM activation induced by CSD. In previous study, cortical application of KCl failed to activate the TRN when rats were anaesthetized as well [25]. The thalamus transmits sensory information between the peripheral nervous system and cortical areas, and participates in pain modulation, consciousness, attention and other cognitive behaviours [31]. The interruption of neuronal processing by anaesthetics may change the mouse’s focused attention from this information disturbance caused by CSD [25], thus playing a protective role in inhibiting the processing of other harmful information.

How is this propagating activation of the VPM induced by CSD? There are several possibilities. Firstly, we found the propagating direction of the VPM activation is related to where CSD was induced, which indicated that this activation may correlate with the synaptic transmission through the projection between the thalamus and the cortex. The thalamus has bidirectional projections into sensory, auditory, visual and other cortical regions [21]. Under normal physiological conditions, thalamus-cortical activity is coordinated in an equilibrium state. The imbalance of integrity caused by cortical functional ablation during CSD may also influence thalamic activity. Clinical studies have demonstrated thalamo-cortical abnormal network dynamics in migraine patients [27], impaired sensorimotor integrity and reduced cortico-cortical inhibition between somatosensory and motor cortices [1]. Thalamic activity can also be altered by a network of GABAergic TRN. It has been demonstrated that CSD can activate electrophysiological activity and c-Fos expression in ipsilateral TRN in awake rats [25]. Previous study on pentobarbital-anaesthetized familial hemiplegic migraine type 1 mutant mice has reported propagation of CSD into the thalamus in S218L HOM mice [11], which may be another possibility. Their study did not find CSD propagating into thalamus in wild-type mice, but it cannot be ruled out because subcortical propagation is more likely to happen in awake state. In our study, VPM also showed no response to CSD in pentobarbital-anaesthetized mice.

As the high-order nucleus of nociceptive afferences, the posterior thalamus nucleus contains dural sensitive neurons and may be closely related to the development of various sensitization reactions, such as photophobia, allodynia, phonophobia and osmophobia [18]. During migraine, thalamus suffers increased low-frequency fluctuation, and its functional connectivity associated with the bilateral TNC was significantly enhanced [30]. Previous study also showed when multiple CSDs were applied in anaesthetized rats, there was a significant increase in activity in trigeminovascular-related brain regions (including TNC, VPM, Po, S1/S2), which reached the maximum amplitude 40 h after CSD induction [10]. CSD can induce long-lasting neuronal activation in meningeal nociceptors and arouse delayed or instant activation of the trigeminal ganglion and the trigeminal nucleus candali (TNC) [32, 33]. However, no significant activation of the ipsilateral thalamus by CSD has been observed [10, 25]. If the VPM response we observed occurs through activation of the trigeminovascular pathway, based on the contralateral projection between the TNC and the thalamus [8], we should have observed a CSD-induced response in the contralateral VPM. However, we only recorded activation of the ipsilateral VPM. One possible reason why we did not observe response in the contralateral VPM is that our imaging field did not cover the VPM area activated by meningeal input. According to the topographic organization of projections from TNC to VPM, the ventrolateral part of the trigeminal spinal nucleus receives mainly ophthalmic afferent endings [29], and projects somatotopic distribution in lateral VPM [13]. However, the GRIN lens was embedded into the intermediate part of the VPM in our studies Besides, in our experiments, KCl was applied over the motor cortex (AP, 2.5 mm; L, 2 mm) which is far from the receptive fields of the dura nociceptors mainly located at the transverse sinus [32]. The application of KCl over motor cortex may not be able to effectively stimulate the nociceptors. Moreover, the increased activity of the thalamus by meningeal stimulation might also be disturbed by the thalamus activity from mouse motion. Our finding provides an indication that the mechanism of the effect we observed is different from the mechanism of migraine pain and other sensitized reactions, such as photophobia. Clinical studies have shown that migraine patients may suffer aura without headache or headache without aura, or both simultaneously [7, 28]. It is suggested that different mechanisms may exist between aura and headache, and the thalamus may have different roles.

Although previous studies have considered CSD to be a potential mechanism for spreading auras, the interaction of the thalamo-cortical and cortico-thalamus network makes cortical function and thalamus regulation inseparable [5]. There may be potential correlation between the gradual spreading characteristics of migraine auras and our observation of the spreading-like neuronal response in the ipsilateral VPM. The thalamus sends projections to the majority of cortex, such as the lateral geniculate nucleus and pulvinar to visual cortex, the ventral and dorsal divisions of the medial geniculate nucleus to auditory cortex [22]. These thalamus nuclei may also be influenced by CSD as well as VPM but are not included in our present experiments, and further investigation is needed. Further research is also required to elucidate the exact mechanism underlying the activation of the ipsilateral thalamus during CSD.

## Conclusions

Our findings indicated that CSD can induce propagating activation of the ipsilateral VPM in awake mice. The response might correlate to the cortical location where CSD was induced. This activation could be influenced by anaesthetics. This finding suggests the potential involvement of thalamus in the migraine auras. Nonetheless, further studies are required to demonstrate the mechanisms linking cortex and VPM, such as with chem-genetic manipulation of cortex-VPM circuit.

## Supplementary Information

**Additional file 1.** The dynamic fluorescence changes of ipsilateral VPM neuronal activity during CSD in awake mice. The increase in neural activity in the VPM induced by CSD showed a “propagating-like” spatial pattern that spread from the posterior-lateral to the anterior-medial side of the VPM. The left side showed the original fluorescence signal, the right side showed the relative fluorescence change.

**Additional file 2.** The dynamic fluorescence changes of contralateral VPM neuronal activity during CSD in awake mice. No significant activation of VGluT2 neurons was observed in the VPM after CSD induction in the contralateral cortex. The changed fluorescence signal was highly correlated with the motion of mice.

## Data Availability

The data that support the findings of this study are available from the corresponding author on reasonable request.

## Abbreviations

CSD: cortical spreading depression
VPM: ventral posteromedial nucleus of thalamus
ROI: region of interest
GRIN lens: Gradient Index lens
